# Impact of Race on Decision Making for Nursing Home Residents With Advanced Dementia: Can We Disrupt and Transform?

**DOI:** 10.1093/geroni/igab046.1595

**Published:** 2021-12-17

**Authors:** Ruth Lopez, Ashley Roach, Meghan Hendricksen, Anita Rogers, Fayron Epps, Ellen McCarthy

**Affiliations:** 1 MGH Institute of Health Professions, Boston, Massachusetts, United States; 2 OHSU, Portland, Oregon, United States; 3 Hinda and Arthur Marcus Institute for Aging Research, Boston, Massachusetts, United States; 4 University of Tennessee at Martin, Parsons, Tennessee, United States; 5 Emory University, Atlanta, Georgia, United States; 6 Marcus Institute for Aging Research, Hebrew SeniorLife, Boston, Massachusetts, United States

## Abstract

Despite 20 years of research and numerous experts and associations advocating a palliative approach to care for nursing home (NH) residents with advanced dementia, research consistently demonstrate striking and persistent racial differences in the use of burdensome interventions such as feeding tubes and hospital transfer. Most notable is that Black NH residents experience more burdensome interventions at the end of life. The reasons for these differences are poorly understood. The purpose of this study was to examine NH staff members’ perceptions of advance care planning with proxies of Black and White residents. We conducted thematic analysis of semi-structured interviews with 158 NH staff members gathered as part of the ADVANCE study. This is a large qualitative study in 13 NHs in 4 regions of the country aimed at explaining regional and racial factors influencing feeding tube and hospital transfer rates. We found that NH staff, regardless of region of the country, held several assumptions about Black proxies including: being attached or not wanting to let go; not wanting to talk about death, believing everything must be done; not wanting to play God; having large conflicted families, not trusting; putting on attitude, and tending not to use NHs. We found that these assumptions led some NH staff to feel that rather than engaging in shared decision making, they were engaged in a battle with proxies leading them to pick and choose their battles and at times even giving up trying. Whether these assumptions can be disrupted and transformed will be discussed.

